# Supplementary material for: Barriers and Opportunities for Patient-Reported Outcome Implementation: A National Pediatrician Survey in the United States
Source: Children (Basel). 2022 Feb 2;9(2):185. doi: 10.3390/children9020185 (PMC8870373; doi:10.3390/children9020185)
Supplement: Supplementary file 1 [file children-09-00185-s001.zip › children-1534783-supplementary.pdf]

Supplemental Table S1: Multivariable Analyses for Pediatricians' Demographic, Practice, and Census Region Factors Associated with Human and Logistic Barriers to PRO Assessment

Demo-graphic, practice, and region factors	Time & manpower[1]	Training for using PRO measures[2]	Lengthy PRO measures[3]	Clinically meaningful cut-offs[4]	Available PRO measures[5]	Scoring skills for PROs[6]	Interpret PROs[7]	PRO knowledge[8]	Follow-up & referral[9]	Child's ability[10]	CATs[11]	Payment[12]	PROs improve care[13]	Validity of PRO measures[14]
	OR (95% CI)	OR (95% CI)	OR (95% CI)	OR (95% CI)	OR (95% CI)	OR (95% CI)	OR (95% CI)	OR (95% CI)	OR (95% CI)	OR (95% CI)	OR (95% CI)	OR (95% CI)	OR (95% CI)	OR (95% CI)
**Age**														
20-40	Ref	Ref	Ref	Ref	Ref	Ref	Ref	Ref	Ref	Ref	Ref	Ref	Ref	Ref
≥41	1.97 (0.76, 5.14)	2.30 (0.81, 6.48)	2.08 (0.78, 5.53)	2.21 (0.83, 5.87)	1.65 (0.66, 4.12)	3.02 (1.12, 8.12)*	2.91 (1.09, 7.74)*	2.62 (1.03, 6.72)*	1.82 (0.74, 4.50)	2.15 (0.88, 5.25)	3.37 (1.42, 8.03)**	1.91 (0.89, 4.12)	3.00 (1.30, 6.90)*	3.14 (1.36, 7.24)**
**Sex**														
Male	Ref	Ref	Ref	Ref	Ref	Ref	Ref	Ref	Ref	Ref	Ref	Ref	Ref	Ref
Female	0.91 (0.55, 1.50)	1.11 (0.67, 1.84)	0.97 (0.59, 1.58)	1.12 (0.68, 1.83)	0.78 (0.48, 1.27)	1.07 (0.66, 1.73)	0.98 (0.61M 1,56)	1.10 (0.69, 1.76)	1.52 (0.95, 2.41)	1.11 (0.71, 1.74)	1.50 (0.97, 2.32)	1.13 (0.74, 1.73)	1.32 (0.86, 2.02)	1.55 (1.01, 2.39)*
**Race**														
White	Ref	Ref	Ref	Ref	Ref	Ref	Ref	Ref	Ref	Ref	Ref	Ref	Ref	Ref
Other	0.87 (0.51, 1.48)	1.08 (0.63, 1.86)	0.93 (0.55, 1.57)	1.21 (0.71, 2.06)	1.27 (0.75, 2.15)	1.08 (0.65, 1.81)	1.02 (0.62, 1,68)	1.26 (0.76, 2.10)	1.80 (1,06, 3.04)*	9.60 (0.60, 1.54)	1.66 (1.03, 2.65)*	0.91 (0.58, 1.43)	1.04 (0.66, 1.65)	1.02 (0.64, 1.61)
**Specialty**†														
GP	Ref	Ref	Ref	Ref	Ref	Ref	Ref	Ref	Ref	Ref	Ref	Ref	Ref	Ref

C/P	1.05 (0.55, 2.00)	0.85 (0.46, 1.58)	1.04 (0.56, 1.96)	1.07 (0.57, 1.99)	1.19 (0.63, 2.25)	0.80 (0.44, 1.44)	0.94 (0.52, 1.70)	0.46 (0.26, 0.83)**	0.89 (0.49, 1.62)	1.12 (0.62, 2.02)	1.10 (0.63, 1.93)	0.66 (0.38, 1.14)	0.97 (0.56, 1.69)	1.03 (0.59, 1.80)
E/R	1.16 (0.48, 2.80)	1.57 (0.62, 4.01)	1.07 (0.45, 2.53)	0.81 (0.36, 1.82)	0.90 (0.39, 2.07)	1.13 (0.49, 2.60)	0.83 (0.34, 1.82)	0.54 (0.25, 1.17)	0.97 (0.43, 2.16)	0.91 (0.42, 2.00)	1.18 (0.55, 2.53)	1.38 (0.65, 2.93)	1.33 (0.63, 2.82)	0.87 (0.41, 1.84)
HO	1.16 (0.54, 2.50)	1.75 (0.78, 3.92)	1.07 (0.51, 2.24)	1.41 (0.66, 3.00)	0.82 (0.40, 1.68)	1.34 (0.64, 2.81)	1.47 (0.70, 3.08)	0.85 (0.41, 1.75)	1.01 (0.50, 2.02)	0.92 (0.46, 1.82)	0.94 (0.49, 1.82)	0.74 (0.39, 1.39)	0.83 (0.43, 1.60)	1.05 (0.54, 2.02)
G/N	2.01 (0.76, 5.30)	1.85 (0.73, 4.66)	1.34 (0.57, 3.15)	2.59 (0.99, 6.81)	1.34 (0.57, 3.15)	2.24 (0.89, 5.61)	1.59 (0.70, 3.64)	1.43 (0.59, 3.47)	0.86 (0.40, 1.86)	0.58 (0.28, 1.19)	1.04 (0.50, 2.15)	1.77 (0.84, 3.74)	1.10 (0.53, 2.25)	0.99 (0.48, 2.04)
**Practice duration**														
0-10	Ref	Ref	Ref	Ref	Ref	Ref	Ref	Ref	Ref	Ref	Ref	Ref	Ref	Ref
≥11	0.59 (0.23, 1.51)	0.30 (0.11, 0.83)*	0.42 (0.16, 1.10)	0.49 (0.19, 1.28)	0.68 (0.85, 3.63)	0.38 (0.14, 1.00)	0.37 (0.14, 0.98)*	0.34 (1.02, 3.98)*	0.50 (0.20, 1.22)	0.53 (0.22, 1.27)	0.48 (0.20, 1.13)	0.89 (0.95, 3.50)	2.92 (0.23, 1.16)	0.52 (0.23, 1.19)
**Practice setting**														
Academic	Ref	Ref	Ref	Ref	Ref	Ref	Ref	Ref	Ref	Ref	Ref	Ref	Ref	Ref
Private	0.90 (0.52, 1.57)	1.49 (0.85, 2.61)	0.98 (0.57, 1.69)	1.25 (0.72, 2.17)	0.81 (0.47, 1.38)	1.24 (0.73, 2.11)	1.23 (0.73, 2.07)	0.87 (0.52, 1.46)	1.22 (0.73, 2.04)	0.91 (0.55, 1.50)	1.00 (0.62, 1.63)	1.14 (0.71, 1.83)	1.30 (0.81, 2.10)	1.34 (0.83, 2.17)
**Census**														

**region**														
South	2.39 (1,19, 5.15)*	1.79 (0.87, 3.70)	2.51 (1.22, 5.16)*	2.32 (1.14, 4.73)*	1.76 (0.85, 3.63)	1.88 (0.92, 3.81)	2.22 (1.12, 4.37)*	2.02 (1.02, 3.98)*	2.13 (1.06, 4.28)*	2.16 (1.10, 4.26)*	1.23 (0.64, 2.39)	1.83 (0.95, 3.50)	2.92 (1.50, 5.68)**	3.06 (1.55, 6.06)**
Northeast	2.11 (1.00, 4.46)	1.62 (0.80, 3.28)	1.97 (0.99, 3.91)	1.94 (0.97, 3.86)	1.27 (0.64, 2.51)	1.26 (0.65, 2.45)	1.79 (0.93, 3.43)	1.57 (0.81, 3.01)	1.30 (0.67, 2.49)	0.95 (0.50, 1.78)	0.45 (0.23, 0.85)*	1.56 (0.83, 2.95)	1.67 (0.87, 3.21)	2.32 (1.19, 4.54)*
Midwest	1.41 (0.75, 2.66)	2.21 (1.14, 4.29)*	2.22 (1.19, 4.15)*	2.58 (1.36, 4.87)**	1.67 (0.88, 3.14)	2.32 (1.23, 4.39)**	2.86 (1.54, 5.31)**	2.33 (1.27, 4.28)**	1.90 (1.03, 3.50)*	2.10 (1.15, 3.82)*	1.21 (0.67, 2.20)	1.51 (0.85, 2.70)	3.15 (1.72, 5.76)	3.40 (1.83, 6.33)***
West	Ref	Ref	Ref	Ref	Ref	Ref	Ref	Ref	Ref	Ref	Ref	Ref	Ref	Ref

OR = odds ratio; CI = confidence interval; NS = variables not selected into the final mode per stepwise approach (p-value ≥0.2); Ref = reference group.

* p-value <0.05; ** p-value <0.01; *** p-value <0.001

† Specialty: GP = General Pediatrics; C/P = Pediatric Cardiology or Pulmonology; E/R = Pediatric Endocrinology or Rheumatology; HO = Pediatric Hematology Oncology; G/N = Pediatric Gastroenterology or Nephrology

1. Limited time and manpower for assessing PROs
2. Limited training on how to administer PRO instruments
3. Long length of PRO instruments
4. Lack of clinically meaningful cut-offs for PRO scores
5. Unavailability of appropriate PRO instruments
6. Limited skills on scoring PRO results
7. Limited ability to interpret PRO results
8. Limited knowledge of PRO concepts
9. Lack of recommendations on follow-up and referral services
10. Varying capabilities of children
11. Unavailability of computerized mode for administering PROs
12. Lack of reimbursement incentives for assessing PROs
13. Lack of evidence that PRO assessment improves care
14. Skepticism about the validity of PRO instruments

Supplemental Table S2: Multivariable Analyses for Pediatricians' Demographic, Practice, and Census Region Factors Associated with Confidence in PRO Assessment

Demographic, practice and region factors	Benefit of PRO assessment[1]	Compatible with the norm[2]	Ability to administer PRO measures[3]	Available PRO measures[4]
	**OR (95% CI)**	**OR (95% CI)**	**OR (95% CI)**	**OR (95% CI)**
**Age**				
20-40	Ref	Ref	Ref	Ref
≥41	0.72 (0.33, 1.55)	0.66 (0.30, 1.47)	0.94 (0.31, 2.29)	1.36 (0.51, 3.43)
**Sex**				
Male	Ref	Ref	Ref	Ref
Female	0.85 (0.56, 1.29)	1.20 (0.79, 1.84)	0.87 (0.55, 1.38)	0.95 (0.57, 1.57)
**Race**				
White	Ref	Ref	Ref	Ref
Other	1.60 (1.03, 2.49)*	1.01 (0.64, 1.60)	1.11 (0.67, 1.82)	1.47 (0.87, 2.50)
**Specialty**				
GP	Ref	Ref	Ref	Ref
C/P	1.02 (0.60, 1.75)	1.00 (0.57, 1.72)	1.29 (0.71, 2.33)	1.29 (0.67, 2.51)
E/R	0.46 (0.21, 1.02)	0.90 (0.42, 1.92)	1.14 (0.51, 2.58)	1.20 (0.48, 3.00)
HO	1.41 (0.75, 2.63)	1.58 (0.84, 2.97)	0.89 (0.43, 1.83)	1.76 (0.84, 2.51)
G/N	1.13 (0.57, 2.25)	0.74 (0.36, 1.52)	0.91 (0.41, 2.02)	1.05 (0.55, 2.51)
**Practice duration**				
0-10	Ref	Ref	Ref	Ref
≥11	1.34 (0.63, 2.86)	1.63 (0.74, 3.58)	1.61 (0.67, 3.87)	0.88 (0.35, 2.18)
**Practice setting**				
Academic	Ref	Ref	Ref	Ref
Private	0.59 (0.37, 0.94)*	0.42 (0.26, 0,68)***	0.72 (0.43, 1.21)	0.74 (0.41, 1.31)

**Census region**				
South	0.77 (0.41, 1.44)	1.05 (0.55, 2.00)	0.71 (0.36, 1.42)	0.60 (0.29, 1.24)
Northeast	0.85 (0.46, 1.59)	1.27 (0.67, 2.40)	0.99 (0.51, 1.93)	0.78 (0.39, 1.58)
Midwest	0.76 (0.43, 1.33)	0.96 (0.53, 1.71)	0.68 (0.37, 1.28)	0.50 (0.26, 1.00)
West	Ref	Ref	Ref	Ref

OR = odds ratio; CI = confidence internval; NS = variables not selected into the final mode per stepwise approach (p-value ≥0.2); Ref = reference group.

* p-value <0.05; ** p-value <0.01; *** p-value <0.001

† Specialty: GP = General Pediatrics; C/P = Pediatric Cardiology or Pulmonology; E/R = Pediatric Endocrinology or Rheumatology; HO = Pediatric Hematology Oncology; G/N = Pediatric Gastroenterology or Nephrology

1. More benefits of PRO assessment than clinical judgments alone
2. PRO assessment compatible with my norms
3. Abilities to administer PRO instruments
4. Available instruments accurately evaluate PROs
